# Cultivation and utility of *Piptoporus betulinus* fruiting bodies as a source of anticancer agents

**DOI:** 10.1007/s11274-016-2114-4

**Published:** 2016-07-27

**Authors:** Małgorzata Pleszczyńska, Adrian Wiater, Marek Siwulski, Marta K. Lemieszek, Justyna Kunaszewska, Józef Kaczor, Wojciech Rzeski, Grzegorz Janusz, Janusz Szczodrak

**Affiliations:** 1 Department of Industrial Microbiology, Maria Curie-Skłodowska University, Akademicka 19, 20-033 Lublin, Poland; 2 Department of Medical Biology, Institute of Rural Health, Jaczewskiego 2, 20-095 Lublin, Poland; 3 Department of Virology and Immunology, Maria Curie-Skłodowska University, Akademicka 19, 20-033 Lublin, Poland; 4 Department of Biochemistry, Maria Curie-Skłodowska University, Akademicka 19, 20-033 Lublin, Poland; 5 Department of Vegetable Crops, Poznań University of Life Sciences, Dąbrowskiego 159, 60-594 Poznan, Poland

**Keywords:** Anticancer activity, Cultivation, Fruiting body, *Piptoporus betulinus*

## Abstract

*Piptoporus betulinus* is a wood-rotting basidiomycete used in medicine and biotechnology. However, to date, no indoor method for cultivation of this mushroom fruiting bodies has been developed. Here we present the first report of successful production of *P. betulinus* mature fruiting bodies in artificial conditions. Four *P. betulinus* strains were isolated from natural habitats and their mycelia were inoculated into birch sawdust substrate supplemented with organic additives. All the strains effectively colonized the medium but only one of them produced fruiting bodies. Moisture and organic supplementation of the substrate significantly determined the fruiting process. The biological efficiency of the *P. betulinus* PB01 strain cultivated on optimal substrate (moisture and organic substance content of 55 and 65 and 25 or 35 %, respectively) ranged from 12 to 16 %. The mature fruiting bodies reached weight in the range from 50 to 120 g. Anticancer properties of water and ethanol extracts isolated from both cultured and nature-derived fruiting bodies of *P. betulinus* were examined in human colon adenocarcinoma, human lung carcinoma and human breast cancer cell lines. The studies revealed antiproliferative and antimigrative properties of all the investigated extracts. Nevertheless the most pronounced effects demonstrated the ethanol extracts, obtained from fruiting bodies of cultured *P. betulinus*. Summarizing, our studies proved that *P. betulinus* can be induced to fruit in indoor artificial culture and the cultured fruiting bodies can be used as a source of potential anticancer agents. In this respect, they are at least as valuable as those sourced from nature.

## Introduction

*Piptoporus betulinus* (Bull.: Fr.) P. Karst. (*Basidiomycota*, *Agaricomycetes*, *Polyporales*, *Fomitopsidaceae*) is a common and important parasite of various birch species in Europe, North America, and Asia. It causes a brown rot of wood of old and weakened trees. *P. betulinus* is a potentially edible mushroom, commonly known as the Birch bracket, Birch polypore, or Razor strop. It forms annual white to brownish fruiting bodies on birch trunks and branches (Stamets 2000).

The medicinal properties of the species have long been used in folk medicine. Infusions of its fruiting bodies were supposed to have strengthening and soothing activity. The species was also regarded as an antibacterial, antiparasitic, and laxative agent and applied in wound healing and as an adjuvant in treatment of rectal cancer and stomach diseases (Grienke et al. 2014; Peintner and Pöder 2000). As shown by pharmacological studies, the Birch polypore can also be widely used in modern medicine. The extracts of *P. betulinus* exhibited various biological activities, mainly cytotoxic and anti-proliferative, against cancer cells (Cyranka et al. 2011; Kandefer-Szerszeń and Kawecki 1974; Lemieszek et al. 2009). Many bioactive secondary metabolites, especially triterpenoids, were also isolated and identified (Grienke et al. 2014; Wangun et al. 2004). The examples are polyporenic acids with anti-inflammatory activities (Kamo et al. 2003) which, together with a hydroquinone, another compound isolated from *P. betulinus*, were additionally identified as matrix metalloproteinase inhibitors (Kawagishi et al. 2002). Furthermore, Schlegel et al. (2000) reported antimicrobial activity of *P. betulinus* extracts that was related to a N-containing compound, piptamine, acting especially against *Staphylococcus aureus* and *Enterococcus faecalis* as well as showing hemolytic activity.

The cell wall of the fungus also contains important polysaccharides, i.e. glucans (Grün 2003; Jelsma and Kreger 1979; Olennikov et al. 2012). Wiater et al. (2011) isolated and characterized water-insoluble, alkali-soluble (1 → 3)-α-D-glucan and demonstrated that its carboxymethylated form had cytotoxic or mitochondrial metabolism-modulating effects. A new, non-medical use of the Birch polypore is also associated with the cell wall (1 → 3)-α-D-glucans. It has been shown that these polymers effectively induce the production of microbial (1 → 3)-α-glucanases, enzymes that have potential in dental caries prevention. The fungal glucans can replace in this role a previous glucanase inducer, streptococcal (1 → 3),(1 → 6)-α-D-glucan which is expensive and hard to reach (Wiater et al. 2008).

Isolation of bioactive ingredients from mushrooms requires collection of large amounts of biological material. However, the accessibility of *P. betulinus* fruiting bodies is limited; the fungus occurs seasonally only in the northern hemisphere, exclusively on birch trees. Cultivation of *P. betulinus* in artificially controlled conditions could provide a standardized material, free of biotic and abiotic contaminants, regardless of the season and geographical location.

To the best of our knowledge, no work has been published on the indoor cultivation of *P. betulinus* fruiting bodies. In this paper we report the production of fruiting bodies of *P. betulinus* on sawdust substrate. Furthermore, since *P. betulinus* is well known for its medicinal properties including anticancer activities, we examined the possibility of obtaining chemopreventive agents from its fruiting bodies cultivated under artificial conditions. We also tested if the anticancer activity of the mentioned extracts was comparable to the properties of analogous extracts obtained from wild growing fruiting bodies of the fungus.

## Materials and methods

### Strains

The carpophores of four *P. betulinus* strains were collected from *Betula pendula* Roth. trees in some regions of Poland in 2008. The strains are marked as PB01, PB02, PB03, and PB04. Pure cultures of the investigated strains were obtained by excising pieces of trama from inner parts of carpophores and transferring them onto malt extract agar medium and then onto potato dextrose agar medium. Pure cultures were deposited at the Collection of Edible and Medicinal Mushrooms of the Department of Vegetable Crops of Poznań University of Life Sciences. Mycelium for inoculation of cultivation substrates was prepared on wheat grains using the commonly known method (Stamets 2000).

### Identification of the *Piptoporus* species using ribosomal internal transcribed spacer (ITS) regions of nuclear ribosomal DNA

The DNA extraction procedure followed the methods of Borges et al. (1990) with minor modifications. The purity and concentration of the DNA samples were evaluated using ND-1000 (Nanodrop, USA). Polymerase chain reaction amplifications (PCR) followed the protocol of White et al. (1990). The primers ITS1 and ITS4 were used for PCR amplification and sequencing of the internal transcribed spacers from the ribosomal genes. Reactions were performed in a TPersonal thermocycler (Biometra, Germany). Amplified PCR products were quantified by gel electrophoresis on a 1 % agarose gel stained with ethidium bromide and purified by microfiltration using a Clean-up kit (A&A Biotechnology, Poland). Sequencing was performed by fluorescent dye-terminator chemistry with the automated sequencer ABI 3730 (Applied Biosystems Inc., USA) following the manufacturer’s instructions. Sequencing data were analyzed with Lasergene v. 8.0 software (DNASTAR, Inc). Database searches were performed with the BLAST and FASTA programs at the National Centre for Biotechnology Information (Bethesda, MD, USA) and the European Bioinformatics Institute (Hinxton, UK).

### Cultivation experiment

A mixture of fresh birch sawdust (65 % v/v) and dry birch chips (35 % v/v) was used as a basal substrate component for the substrate preparation to produce *P. betulinus* fruiting bodies. Each substrate portion was supplemented with a mineral additive including (g/bag) gypsum, 50; dolomite, 20, and sucrose, 15. The medium was also enriched with an organic supplement containing (% dry weight (dw/dw) wheat bran, 35; rye bran, 20; ground corn, 15; soybean powder, 15; rye grain, 10; and millet grain, 5. To determine the optimum content of organic substances in the substrate, 15, 25, 35, or 45 % (dw/dw of the substrate) of the organic mixture were added. Simultaneously, the moisture of the substrates was also optimized by adjusting the water content to 45, 50, 55, 60, or 65 %. The prepared substrates were placed in polypropylene bags with microporous filters (Mycomed, Poland). Each bag of 22 cm × 12 cm × 17 cm in size contained 1.4 kg dry weight of the substrate. The bags with the substrates were sterilized for 8 h at 105–108 °C, then cooled down to 20 °C, and inoculated with 20 g grain spawn per bag. Tightly closed bags were subsequently kept in a spawn running room, at 23 ± 1 °C and air humidity of 65–70 %. Incubation was continued until the entire surface of the substrate was colonized by the mycelium.

Following the mycelium run, the cultures were subjected to cold shock (2–4 °C for 48 h) and then kept at 16–18 °C and humidity of 80–85 % under fluorescent light illumination of 200–250 lux for 10 h/day. Adequate ventilation was provided to prevent an increase in the CO_2_ concentration above 1000 ppm. The fruiting bodies were harvested when mature. Biological efficiency (BE %) was calculated using an equation reported by Stamets (2000) as follows: (fresh weight of harvested mushrooms/dry matter content of the substrate) × 100.

### Evaluation of anticancer properties of ethanol and water extracts isolated from *P. betulinus* fruiting bodies in carcinoma cell culture model

#### Extract preparation

Fruiting bodies of *P. betulinus* collected from nature in 2008 or cultivated in 2009 and 2010 (two batches of PB01 fruiting bodies) were dried and comminuted using a laboratory mill. One part of each of the powder obtained was extracted with ethanol for a few days in Soxhlet apparatus, while another one was suspended in water and shaken for a few days at room temperature. Both ethanol and water solutions were concentrated by evaporation and vacuum drying to dark brown extracts. For artificial cultures of *P. betulinus*, the extraction yields were 20.3 % (ethanol extract from the first batch of the fruiting bodies, marked as PB-A1e), 18.2 % (ethanol extract from the second batch, PB-A2e), 15.0 % (water extract, PB-A1w), and 25.2 % (water extract, PB-A2w). For wild *P. betulinus*, the extraction yields were 20.1 % (ethanol extract, PB-Ne) and 13.0 % (water extract, PB-Nw).

Ethanol and water extract stock solutions were prepared in DMSO (100 mg/ml) or PBS (10 mg/ml), respectively. Working solutions were prepared by dissolving an appropriate stock solution in culture medium.

#### Cell lines

The human lung carcinoma (A549), human colon adenocarcinoma (HT-29) and human breast cancer (T47D) cell lines were obtained from ECACC (European Collection of Cell Cultures, Centre for Applied Microbiology and Research, Salisbury, UK). The cells were kept in the following culture media purchased from Sigma (Sigma Chemicals, St. Louis, MO, USA): A549—3:1 mixture of DMEM and Nutrient mixture F-12 Ham; HT-29 and T47D—1:1 mixture of DMEM and Nutrient mixture F-12 Ham. All media were supplemented with fetal bovine serum (FBS, 10 %) (Sigma), penicillin (100 U/ml) (Sigma) and streptomycin (100 μg/ml) (Sigma). The cells were maintained in a humidified atmosphere of 95 % air and 5 % CO_2_ at 37 °C.

#### MTT assay: cell proliferation assessment

A549, T47D and HT-29 cells were seeded on 96-well microplates at a density of 1 × 10^4^ cells/ml, 2 × 10^4^ cells/ml and 3 × 10^4^ cells/ml, respectively. The next day, the culture medium was removed and the cells were exposed to serial dilutions of the extracts, with concentrations ranging from 0 to 250 μg/ml, prepared in fresh medium supplemented with FBS. Cell proliferation was assessed after 96 h of incubation under standard conditions (5 % CO_2_, 37 °C) by means of the MTT assay, in which the yellow tetrazolium salt (MTT) is metabolized by viable cells to purple formazan crystals. After the incubation period, an MTT solution (5 mg/ml in PBS) was added to the cells for 3 h. Resultant crystals were solubilized overnight in SDS buffer, pH 7.4 [10 % SDS (Sigma) in 0.01 M HCl], and the product was quantified spectrophotometrically by measuring the absorbance at 570 nm wavelength using an E-max Microplate Reader (BioTek ELx800, Highland Park, Winooski, VT, USA).

#### Wound assay: cell migration assessment

A549, HT-29 and T47D cells were plated at 2 × 10^5^ cells on 3-cm culture dishes (Nunc). The next day, the cell monolayer was scratched by a pipette tip (P300) and the number of cells that had migrated into the wound area after 24 h in the absence (control) or presence of ethanol (50 μg/ml) or water extracts (250 μg/ml) was estimated. The cells were stained with the May–Grünwald–Giemsa method. Microscopic analysis was performed with an Olympus BX51 System Microscope (Olympus Optical CO., LTD, Tokyo, Japan) and micrographs were prepared in analySIS software (Soft Imaging System GmbH, Münster, Germany). Cells that migrated to the wound area were counted on the micrographs and the results were expressed as a mean cell number that had migrated to the wound areas taken from 4 micrographs.

### Statistical analysis

In the cultivation experiment, five replications were carried out for each combination of the strain, substrate moisture, and organic supplement content. The data were presented as the mean value and standard error of the mean (SEM). Statistical analysis of the results obtained from the in vitro experiments was performed using one way-ANOVA with the Tukey post hoc test and column statistics used for comparisons. Significance was accepted at *p* < 0.05. The IC_50_ value (a concentration causing 50 % inhibition of proliferation, compared to the control) was calculated according to the Litchfield and Wilcoxon method (1949).

## Results

### Production of *P. betulinus* fruiting bodies

The isolated strains of *Piptoporus* were identified at the species level by analysis of their ITS regions. For each isolate, one product ranging from 661 bp (PB04) to 663 bp (PB01) in length was obtained from PCR with ITS1 and ITS4 primers. The complete sequences of these products indicated from 99 to 100 % identity to the *P. betulinus* ITS sequences. The nucleotide sequences have been deposited in the GenBank nucleotide sequence database under Accession No. from KT207811 to KT207814.

**Fig. 1:**
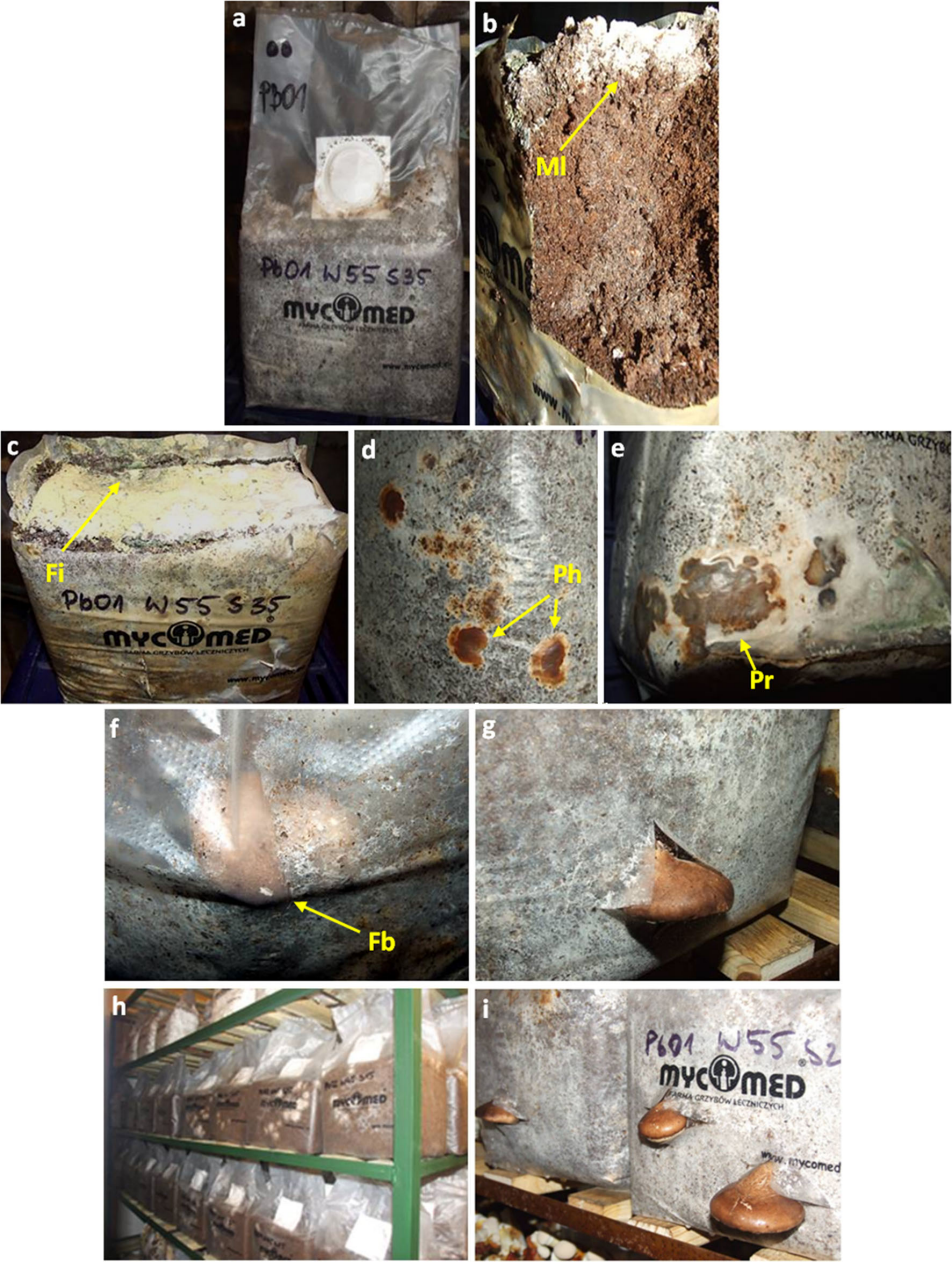
Formation of a *P. betulinus* PB01 fruiting body on birch sawdust. **a** Bag with growing mycelium (20 days after inoculation). **b** Longitudinal section of the substrate overgrown by mycelium. **c** Infection of the substrate surface with *Trichoderma* sp. **d** 9-day-old pinheads. **e** 20-day primordia. **f** Fruiting body inside the bag. **g** Mature fruiting body. **h** Bags with growing mycelium. **i** Bags with mature fruiting bodies. *Ml* mycelium layer, *Fi* fungal (*Trichoderma*) infection, *Ph* pinheads, *Pr* primordium, *Fb* fruiting body

Birch sawdust was used as a basal substrate for growth of *P. betulinus.* It was enriched with a constant concentration of a mineral additive and an organic supplement used in the concentration range from 15 to 45 %. The moisture content of the media tested ranged from 45 to 65 %. Sterilized media were inoculated with four isolated strains of *P. betulinus* and then incubated until complete colonization by the mycelium (Fig. 1a). Generally, no correlations between the duration and efficiency of the colonization process and the substrate moisture and organic supplementation were observed. The only noticeable factor that slightly differentiated the colonization process was the Birch polypore strains used. The substrates were colonized the fastest by the mycelium of strain PB01 (data not shown). However, after 28 days of incubation, a vast majority of the substrate cubes were completely overgrown by *P. betulinus* mycelium. A uniform, greyish mycelium layer with a thickness not exceeding 1 mm formed on the surface of the substrate. Under the layer, a dark brown substrate with whitish mycelium hyphae was seen. After removing the polypropylene bag, the cube formed admittedly did not fall apart but it was not also a compact structure so it was easy to break into pieces at careless handling (Fig. 1b).

The cold shock was effective in triggering the formation of *P. betulinus* primordia. The cubes of the substrate were subjected to cold shock without opening the bag, due to the risk of infections by fungi of the genus *Trichoderma* and *Penicillium* (Fig. 1c), and then placed in conditions optimal for fruiting. Pinheads could be observed under the foil, inside the bag, already within 8–10 days after induction (Fig. 1c), and then they began to develop into fruiting bodies (Fig. 1d–f). However, the PB01 was the only strain that produced mature fruiting bodies. In some cases, incision of the bag to release the forming fruiting body stopped its growth and resulted in death of the primordium, and then development of pathogenic infection.

**Fig. 2:**
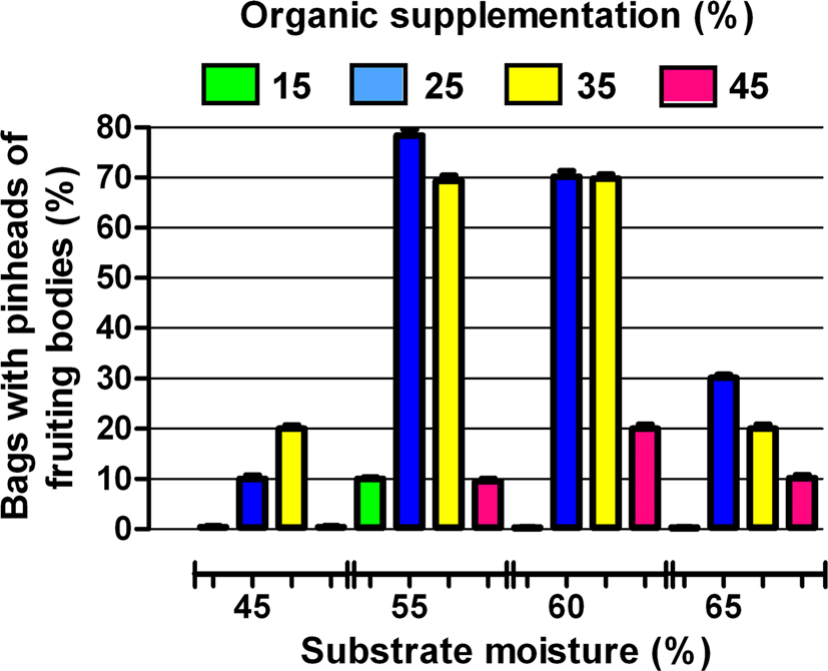
Effects of organic supplementation and moisture of the substrate on formation of fruiting bodies by *P. betulinus* PB01

The moisture of the medium and the organic supplementation significantly determined the fruiting process (Fig. 2). Most frequently, fruiting bodies appeared when *P. betulinus* was cultivated on substrates with moisture content of 55 and 65 % and simultaneously containing 25 or 35 % of organic substances. In these conditions, within 30–45 days, one flush of fruiting bodies having a diameter of 7–9 cm and reaching a weight of 50–120 g was obtained.

**Fig. 3:**
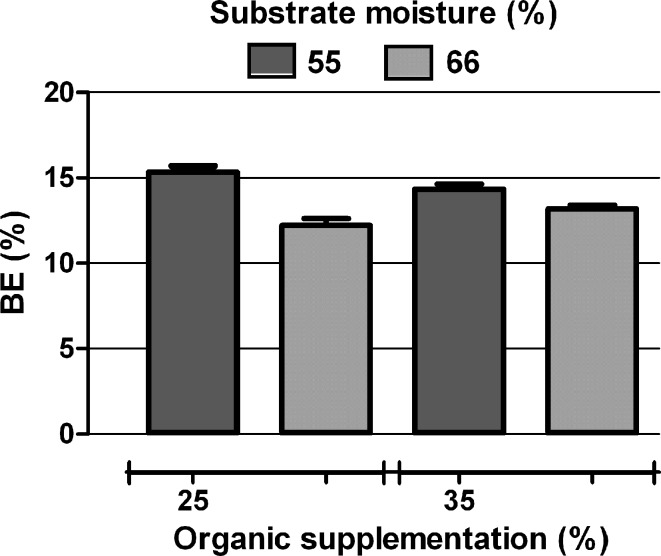
Biological efficiency of *P. betulinus* PB01 cultivated on substrates containing an optimal amount of water and organic additive

The biological efficiency (BE) calculated for fungi growing in the bags containing substrate with an optimal composition ranged from 12 to 16 % (Fig. 3). The moderate weight of the harvested fruiting bodies and BE value resulted from the specific structure of the fungal mat and infections at the carpophore fracture site.

### Anticancer activities of the extracts from fruiting bodies of wild and cultured *P. betulinus*

**Fig. 4:**
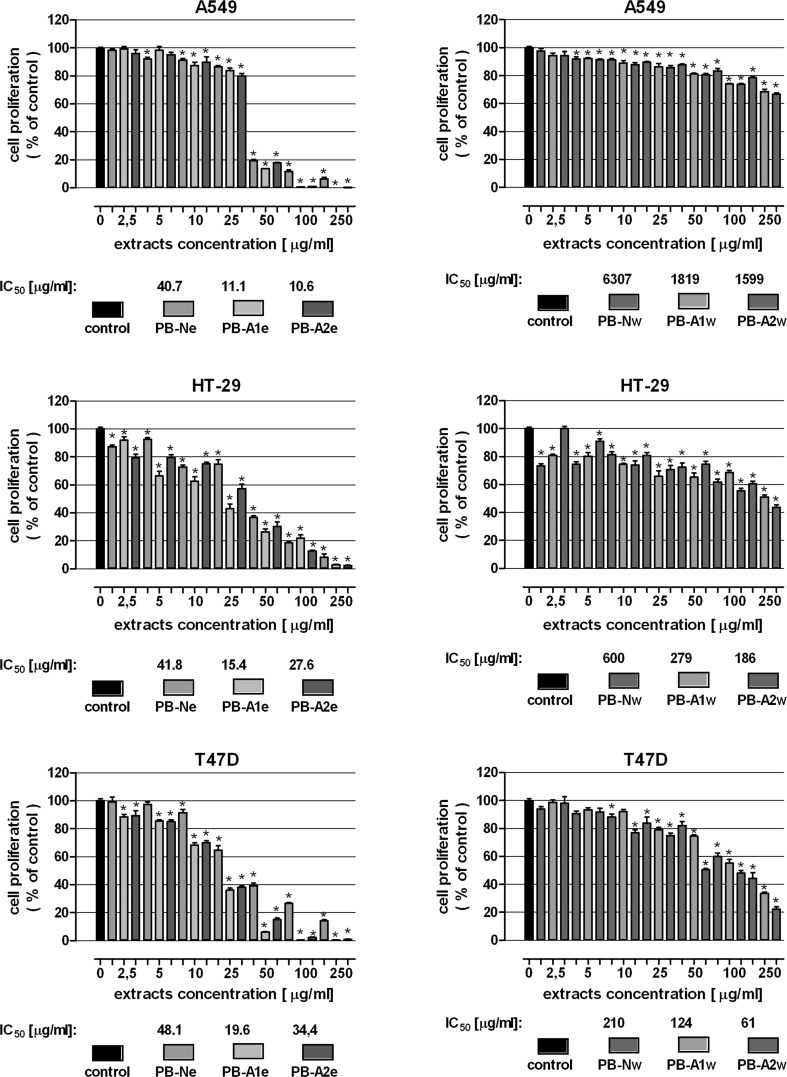
Antiproliferative effect of extracts isolated from *P. betulinus* in human lung carcinoma (A549), human colon adenocarcinoma (HT-29) and human breast cancer (T47D) cell lines. Cells were incubated with ethanol (*left panel*) and water extracts (*right panel*) in concentrations ranging from 0 to 250 μg/ml for 96 h. Cell proliferation was measured by the MTT assay. Results are presented as the mean ± SEM of 6 measurements. *p < 0.05 versus control, one-way ANOVA test; post test: Tukey

The anticancer activities of the water and ethanol extracts obtained from the fruiting bodies of cultured and wild *P. betulinus* was verified in human lung carcinoma (A549), human colon adenocarcinoma (HT-29) and human breast cancer (T47D) cell lines. In the first set of experiments, the antiproliferative activity of mushroom extracts was assessed. A549, HT-29 and T47D cells were subjected to increasing concentrations of the tested extracts and cell proliferation was examined by the MTT assay. As presented in Fig. 4, all the tested extracts inhibited proliferation of cancer cells in dose-dependent manner. The most pronounced antiproliferative effect was shown for the ethanol extracts prepared from *P. betulinus* artificial cultures: the IC_50_ value (a concentration causing proliferation inhibition by 50 % compared to control) were in ranges 11.1–19.6 µg/ml (PB-A1e) and 10.6–34.4 µg/ml (PB-A2e). On the other hand the ethanol extract of fruiting bodies collected in forest (PB-Ne) gave slightly worse results; IC_50_ were in ranges 40.7–48.1 µg/ml. It have to be noted that the most sensitive cell lines to ethanol extracts was A549, on the contrary T47D cells were found the most resistant.

In contrast to the ethanol extracts, the water extracts induced much weaker inhibition of cancer cell proliferation. Susceptibility of cell line to the treatment was also different compare to the ethanol extracts. The most significant changes were observed in T47D cells: the IC_50_ value amounted to 210 µg/ml (PB-Nw), 124 µg/ml (PB-A1w) and 61 µg/ml (PB-A2w). Instead A549 cells worst reacted to tested extracts: IC_50_ was 6307 µg/ml (PB-Nw), 1818 µg/ml (PB-A1w) and 1599 µg/ml (PB-A2w). Similar to the results obtained from the ethanol extracts, “artificial” extracts (PB-A1w and PB-A2w) inhibited cancer cell proliferation more effectively than the “natural” extract (PB-Nw). Nevertheless in this case PB-A2w revealed better antiproliferative abilities than PB-A1w.

**Fig. 5:**
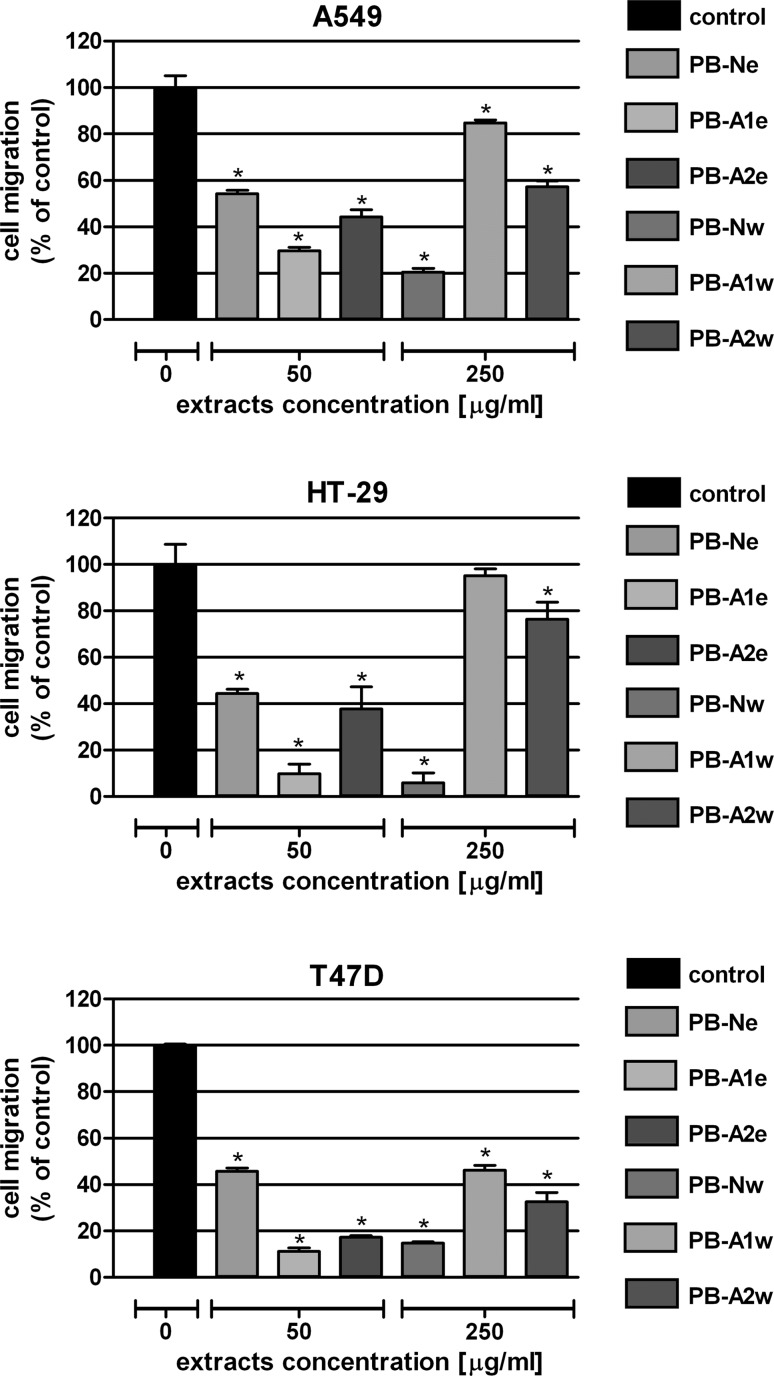
Effect of *P. betulinus* extracts on migration of human lung carcinoma (A549), human colon adenocarcinoma (HT-29) and human breast cancer (T47D) cell lines. Cell motility was analyzed by the wound assay. Scratched monolayers of cells were incubated for 24 h in the absence of extracts (control) or in the presence of ethanol extracts (50 μg/ml) or water extracts (250 μg/ml). Results are expressed as a mean number of cells that had migrated to the wound area ± SEM of 4 measurements. *p < 0.05 versus control, one-way ANOVA test; post test: Tukey

In the next step, the influence of the extracts on colon cancer cell motility was examined by means of the wound assay. A549, HT-29 and T47D cells were exposed for 24 h to the extracts at the chosen concentrations: 50 µg/ml for the ethanol extracts or 250 µg/ml for the water extracts. Microscopic observation revealed greater antimigrative properties of the ethanol extracts than the water extracts (Fig. 5). Among the ethanol extracts, PB-A1e at the concentration of 50 μg/ml was characterized by the most significant inhibition of cancer cells migration; it reduced tested cell motility down to 70.2 % (A549), 90.1 % (HT-29) and 88.9 % (T47D). The results obtained from the water extracts, which were used at a fivefold higher concentration than the ethanol extract, were quite surprising. The most significant antimigrative properties were shown by the PB-Nw extract prepared from *P. betulinus* fruiting bodies collected in the forest. On the contrary, PB-A1w, which was extracted from mushrooms that yielded the most effective ethanol extract, far less affected the cell migration.

## Discussion

To date, for various research uses, *P. betulinus* mycelium has been cultivated in submerged and surface cultures (Cyranka et al. 2011), while the fruiting bodies were obtained only from nature. Production of the fungus fruiting bodies has been described in only one paper. Ka et al. (2008) carried out log cultivation of *P. betulinus* on *Betula davurica.* From one log, they obtained one or two mature fruiting bodies with a weight from 212 to 1298 g during 18-month culture.

In this paper, we reported successful production of *P. betulinus* mature fruiting bodies on a birch sawdust substrate supplemented with organic additives in controlled, artificial conditions. In the experiment, a complete cultivation cycle of *P. betulinus* lasted about 3–4 months and resulted in about 200–250 g fresh mass from one bag of the substrate. Unfortunately, unlike most fungi (Stamets 2000) but similar to another polypore fungus *Laetiporus sulphureus* (Pleszczyńska et al. 2013), *P. betulinus* did not form a dense mycelium mass. For this reason, we failed to obtain larger fruiting bodies or successive harvests of this fungus. Further work is planned to increase both the BE and the quality of fruiting bodies.

*Piptoporus betulinus* has been commonly used in folk medicine as an antiparasitic and antimicrobial agent in the treatment of wounds, and remedy for gastrointestinal disorders (Chang and Wasser 2012; Pöder 2005; Wasser 2010). Anticancer, anti-fatiguing, immunoenhancing, and soothing properties have also been noted (Semerdzieva and Veselsky 1986; Shamtsyan et al. 2004; Reshetnikov et al. 2001). Our previous studies showed anticancer effects of *P. betulinus* water, ethanol, and ether extracts in a variety of tumor cell lines (Kaczor et al. 2004; Lemieszek et al. 2009; Żyła et al. 2005). Water extracts revealed antiproliferative abilities in lung carcinoma, colon adenocarcinoma, and glioma cell cultures (Lemieszek et al. 2009). Ethanol extracts successfully inhibited proliferation and migration of lung cancer cells and T cell leukemia cells (Żyła et al. 2005), while the ether extracts were found as effective killers of neuroblastoma, thyroid carcinoma, breast cancer, epithelial cervical cancer, and laryngeal cancer (Kaczor et al. 2004). However, all the studies mentioned were performed on extracts isolated from fruiting bodies of *P. betulinus* derived from the natural environment. In the present study, we examined the possibility of using fruiting bodies of cultivated *P. betulinus* as a source of compounds with anticancer activity. For this purpose, anticancer activity of water and ethanol extracts from cultivated *P. betulinus* fruiting bodies was examined and compared to the activity of analogous extracts of wild *P. betulinus* fruiting bodies in a variety of cancer cell lines.

The results of the in vitro studies revealed anticancer properties of extracts isolated from cultivated fruiting bodies of *P. betulinus*. Especially the ethanol extract PB-A1e demonstrated particularly preferred impact on investigated cancer cells The described effect was attributed to effective inhibition of both cell proliferation and migration of human lung carcinoma, human colon adenocarcinoma and human breast cancer cell lines. After successful completion of additional analyses including cytotoxicity examination, this extract could be considered for further in vivo studies.

Mushrooms, the fruiting bodies of basidiomycetous fungi, contain many valuable substances for medical applications. Their potential, however, is not fully exploited, given the fact that fruiting bodies of a majority of species cannot be cultured. An unsolved problem in the science of medicinal mushrooms is also the variability of biological material collected from nature. The presented results showed comparable anticancer activity of extracts isolated from cultivated and naturally grown *P. betulinus* mushroom. Controlled cultivation of *P. betulinus* can facilitate research (inter alia, by enhancing the accessibility of the fungal material) and allow standardized production of mushroom-based safe mycopharmaceuticals.
